# Expert evaluation of GPT-4o and Gemini responses to patient questions on carotid endarterectomy

**DOI:** 10.1590/1806-9282.20251453

**Published:** 2026-05-01

**Authors:** Ömer Faruk Rahman, Alper Özbakkaloğlu, Mert Arslangilay, Ahmet Daylan, Ercan Keleş, Önder Turgut Bozkurt, Şahin Bozok

**Affiliations:** 1 İzmir Bakırçay University, Department of Cardiovascular Surgery – İzmir, Turkey.; 2 İzmir Çiğli Training and Research Hospital, Department of Cardiovascular Surgery – İzmir, Turkey.

**Keywords:** Artificial intelligence, Carotid endarterectomy, Health communication, Large language models, Patient education

## Abstract

**OBJECTIVE:**

The aim of this study was to compare the accuracy, scientific quality, and clarity of responses generated by GPT-4o and Gemini to frequently asked patient questions related to carotid artery disease and carotid endarterectomy.

**METHODS:**

In total, 40 unique carotid endarterectomy-related questions were compiled from online sources and clinical experience. Each was entered into separate new sessions with GPT-4o and Gemini 2.5 Flash in Turkish, and responses were collected without modification. Notably, four blinded cardiovascular surgeons independently rated each answer (1–5 Likert scale) in three domains: Accuracy, Scientific Quality, and Clarity. Mean response lengths and domain scores were compared using appropriate paired tests.

**RESULTS:**

GPT-4o produced longer responses than Gemini (258.1±101.6 vs. 193.2±43.7 words; p<0.001). Overall, GPT-4o had higher Accuracy scores (4.33±0.39 vs. 4.16±0.33; p=0.04), with no significant differences in Scientific Quality or Clarity (p=0.377 and p=0.154, respectively). In rater-level analyses, Gemini scored higher in Clarity for one rater, whereas GPT-4o was superior in Accuracy and Scientific Quality for another. Overall mean scores were comparable (4.17±0.36 vs. 4.13±0.31; p=0.636). Physician referral was recommended in 62.5% of GPT-4o and 52.5% of Gemini (p=0.366).

**CONCLUSION:**

Both GPT-4o and Gemini provided "good"-quality responses to carotid endarterectomy patient questions, with GPT-4o showing a modest accuracy advantage, with no difference in other domains. Explicit disclaimers on both platforms underscore their supportive, not definitive, role in patient education. Physicians should remain the primary source for individualized decisions, and AI-generated information should always be verified.

## INTRODUCTION

Carotid artery disease is a progressive chronic condition that develops on the basis of atherosclerosis and is associated with significant mortality and morbidity. It accounts for approximately 65% of ischemic cerebrovascular disease cases, and its global prevalence has approached 80 million^1^. The ESC 2024 guideline algorithm primarily classifies patients based on the presence of symptoms within the past 6 months^2,3^. Carotid artery disease may become symptomatic with clinical presentations such as transient ischemic attack (TIA), amaurosis fugax, and ischemic stroke, or it may remain asymptomatic. In both asymptomatic and symptomatic patients, the management of risk factors and medical therapy is of great importance. However, in certain clinical scenarios, carotid endarterectomy or carotid artery stenting is also recommended in addition to these therapeutic approaches.

Despite its beneficial outcomes, carotid endarterectomy also carries certain risks. The risk of perioperative complications, such as death or morbid stroke related to carotid endarterectomy, has been reported to range between 3 and 7.5%^4^. This represents a significant concern in the decision-making process for surgery, particularly in individuals with asymptomatic carotid artery disease. Moreover, the increasing adoption of carotid artery stenting as an alternative to surgical treatment may introduce additional uncertainty and confusion for patients. In recent years, patients have relied not only on physicians’ opinions when making healthcare decisions but also on a variety of digital information sources. For many years, patients have relied on the internet for information on diagnosis, treatment, and follow-up. More recently, large language models (LLMs) have become prominent in this role, offering user-friendly interfaces, instant responses, and explanations in the local language.

LLMs explicitly display a warning in their user interfaces stating that they "may generate incorrect or incomplete information." This raises questions about the reliability of the information obtained by patients who make extensive use of online resources. To the best of our knowledge, no prior study has systematically examined responses generated by large language models to patient questions on carotid endarterectomy, particularly through expert evaluation of their accuracy, scientific rigor, and clarity.

To address this gap, our study focused on carotid artery disease, which carries significant morbidity and mortality risk and often involves patient-driven decision-making, and comparatively evaluated the responses generated by GPT-4o and Gemini across three domains through a blinded expert panel.

## METHODS

This study was approved by the Clinical Research Ethics Committee of the Faculty of Medicine, İzmir Bakırçay University, in July 2025 (Approval No: 2382).

### Question set

To construct the question set, local-language keywords describing carotid artery disease were used to comprehensively search Google results, YouTube content, patient forums, and the "Frequently Asked Questions" sections of hospital websites in Turkey. In addition, patient questions frequently encountered in clinical practice were included.

After eliminating duplicate expressions, 40 unique questions were identified, covering the themes of diagnosis–indication (Questions 1–13), operative process–risks (Questions 14–26), and postoperative follow-up–lifestyle (Questions 27–40).

### Large language model response generation and collection

Due to the risk that unauthenticated sessions could redirect the user to different subversions or model variants depending on server traffic, GPT-4o (OpenAI, San Francisco, CA, USA) and Gemini 2.5 Flash (Google DeepMind, Mountain View, CA, USA) were accessed via newly created, free user accounts with no prior interaction history. The 40 questions were entered individually into separate "new chat" sessions for each model and answered in a zero-shot manner, to avoid contextual bias and ensure that prior responses would not influence subsequent ones, without additional system or follow-up prompts. To ensure comparable conditions, all responses were collected within the same session format. The responses were recorded without any modifications, word counts were determined manually, and the mean response lengths of the two models were subjected to statistical analysis.

### Expert panel

The responses generated by the large language models were evaluated by a blinded expert panel consisting of four cardiovascular surgeons from the same institution, each with at least 5 years of clinical experience. The responses were compiled into two anonymized booklets with the model identities concealed; only the data analyst knew which booklet corresponded to which model. The panelists first received the initial booklet and the evaluation form, and the second booklet was delivered at least 7 days later to minimize memory effects (wash-out period). The study designer did not participate in the evaluation process to maintain blinding. For clarity in the analyses, each panelist was labeled as Rater 1 through Rater 4.

Each response was scored on a 1–5 Likert scale for Accuracy, Scientific quality, and Clarity (1="very poor," 2="poor," 3="fair," 4="good," 5="excellent"). The domains were defined as follows and provided to the evaluators in the evaluation form: Accuracy reflected the degree to which the information aligned with current guidelines; Scientific quality indicated the comprehensiveness and level of detail in addressing the topic; and Clarity referred to the ease with which a patient with average health literacy could understand the text.

### Statistical analysis

Given that the sample size in this study was less than 50, the normality of data distribution was assessed using the Shapiro-Wilk test. Paired data with a normal distribution were compared using the paired t-test. The chi-square test was used to compare the frequency of physician referral recommendations between the two models. Quantitative data were reported as mean±standard deviation (SD) or median (min–max), and categorical data were presented as counts and percentages [n (%)]. Statistical significance was set at p<0.05. All analyses were conducted using IBM SPSS Statistics version 26 (IBM Corp., Armonk, NY, USA).

## RESULTS

The mean response length of the GPT-4o model (258.1±101.6 words) was significantly greater than that of the Gemini model (193.2±43.7 words) (p<0.001) (Table 1). When all raters were considered together, GPT-4o received higher scores in the Accuracy domain (4.33±0.39 vs. 4.16±0.33; p=0.04). No statistically significant differences were observed between the models in the Scientific Quality or Clarity domains (p=0.377 and p=0.154, respectively).

**Table 1:** Sample of 20 patient-style questions (out of 40) on carotid artery disease, with GPT-4o and Gemini response word counts and their differences.

Question No.	Question	GPT-4o	Gemini	Difference
1	Can carotid artery occlusion cause fainting?	162	198	36
2	Can carotid artery stenosis cause dizziness?	160	156	-4
3	Which diagnostic test is best for detecting carotid artery narrowing?	395	231	-164
4	Can carotid artery stenosis improve with medication alone?	244	238	-6
5	Which medical specialty performs carotid artery surgery?	89	109	20
6	If surgery is preferred over stenting or medication for carotid stenosis, what is the main reason?	301	221	-80
7	Is the risk of stroke higher if carotid stenosis is monitored without surgery?	333	269	-64
8	Is the stroke risk from carotid surgery higher than from stent placement?	440	218	-222
9	Can both carotid arteries be treated during the same surgery?	300	210	-90
10	At what level of narrowing should carotid artery surgery be considered?	268	171	-97
11	Is surgery performed for asymptomatic carotid stenosis?	365	188	-177
12	Is 100% occluded carotid artery operated on?	354	236	-118
13	After the decision for carotid artery surgery, must I undergo the operation immediately?	306	205	-101
14	In carotid artery surgery, is general anesthesia or local anesthesia preferred?	179	197	18
15	How long is the incision made in carotid artery surgery?	48	113	65
16	How many hours does carotid artery surgery take?	65	91	26
17	Is there a need for blood transfusion during carotid artery surgery?	133	171	38
18	How many hours after carotid artery surgery will I wake up?	176	170	-6
19	Is there a risk of death in carotid artery surgery?	241	189	-52
20	What is the percentage risk of paralysis after carotid artery surgery?	94	178	84
	p-value			<0.001
	Test stat			5.407

p-values were calculated using the paired-samples t-test.

**Figure 1:**
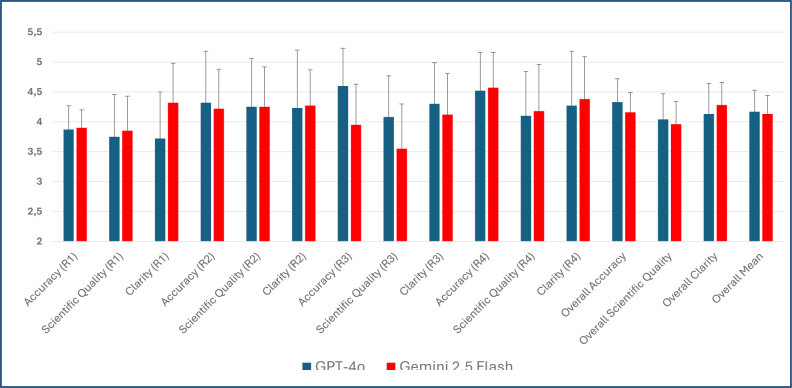
Graphical representation of mean scores (±SD) assigned to GPT-4o and Gemini by four expert raters in three evaluation domains: Accuracy, Scientific Quality, and Clarity.

In rater-level analyses, for Rater 1, a significant difference in favor of Gemini was observed only in the Clarity domain (p<0.001). For Rater 3, GPT-4o was superior in both the Accuracy and Scientific Quality domains (p<0.001). No significant differences were found in the other rater-level analyses. Based on the overall mean scores across all domains, the values were 4.17±0.36 for GPT-4o and 4.13±0.31 for Gemini, with no statistically significant difference between the models (p=0.636) (Table 2). Detailed distributions for each rater and the graphical summary of overall comparisons are presented in Figure 1.

**Table 2:** Comparison of GPT-4o and Gemini scores across three evaluation domains (Accuracy, Scientific Quality, and Clarity) by four expert raters and overall means.

	GPT-4o	Gemini	t-statistic	p-value
Accuracy (R1)	3.87±0.4	3.9±0.3	-0.33	0.743
Scientific quality (R1)	3.75±0.71	3.85±0.58	-0.662	0.512
Clarity (R1)	3.72±0.78	4.32±0.66	-3.972	**<0.001**
Accuracy (R2)	4.32±0.86	4.22±0.66	0.612	0.544
Scientific quality (R2)	4.25±0.81	4.25±0.67	0	1
Clarity (R2)	4.23±0.97	4.27±0.6	-0.28	0.781
Accuracy (R3)	4.6±0.63	3.95±0.68	4.759	**<0.001**
Scientific quality (R3)	4.08±0.69	3.55±0.75	3.787	**<0.001**
Clarity (R3)	4.3±0.69	4.12±0.69	1.312	0.197
Accuracy (R4)	4.52±0.64	4.57±0.59	-0.374	0.711
Scientific quality (R4)	4.1±0.74	4.18±0.78	-0.416	0.68
Clarity (R4)	4.27±0.91	4.38±0.71	-0.561	0.578
Accuracy (overall)	4.33±0.39	4.16±0.33	2.129	**0.04**
Scientific quality (overall)	4.04±0.43	3.96±0.38	0.894	0.377
Clarity (overall)	4.13±0.51	4.28±0.38	-1.455	0.154
Overall mean	4.17±0.36	4.13±0.31	0.476	0.636

R=rater (expert evaluator). p-values were calculated using the paired-samples t-test. Bold values indicate statistically significant differences (p<0.05).

Additionally, the frequency of physician referral recommendations in the responses of both models was analyzed. GPT-4o included statements advising consultation with a physician in 25 out of 40 questions (62.5%), whereas Gemini did so in 21 out of 40 questions (52.5%). However, the difference between these proportions was not statistically significant (p=0.366).

## DISCUSSION

In recent years, LLM-based chatbots have triggered a paradigm shift not only in general information access but also in the healthcare domain. This development represents a second act of transformation, reminiscent of the widespread dissemination of information via the internet in the 1990s. The greatest innovation of AI-powered technologies lies in their ability to provide users with interactive, customizable, and comprehensive information rapidly. A recent systematic review demonstrated that the quality of online health information can directly shape patients’ attitudes and behaviors, whereas low-quality content may lead to adverse outcomes such as poor decision-making, negative emotions, and diminished physician–patient trust^5^. Therefore, the accuracy of the information provided by LLMs is critically important for maintaining the reliability of health communication.

With this objective, our study compared the responses of GPT-4o and Gemini to patient questions regarding carotid artery disease and carotid endarterectomy and evaluated their reliability through an expert panel. To the best of our knowledge, this is the first study to examine responses from different LLMs on these topics using expert assessment. Based on the expert panel's scores, GPT-4o outperformed in the Accuracy domain, while the overall performance of both models remained at a similar level and was rated within the "good" range. This finding is consistent with the study by Patel et al., which examined questions on peripheral artery disease and reported that GPT-4o significantly outperformed Gemini for accuracy, with 70% of its responses rated as "correct"^6^. In the literature, numerous evaluations of LLMs across various clinical fields have suggested that these models generally answer patient questions with ratings of "good" or higher and that, when used with caution, they can provide practically useful content^7–10^. However, some studies have emphasized that the current level of accuracy and source transparency is still insufficient for LLMs to be adopted as a primary source of patient information, and that they cannot replace personalized physician–patient communication^11,12^.

In our study, GPT-4o was found to provide longer responses compared to Gemini. While greater detail may offer richer information, lengthy texts may reduce readability for individuals with low health literacy. Although the mean scores remained similar between the two models, the longer response length may be considered a disadvantage for GPT-4o. Consistent with our findings, studies by Chervonski et al. and Patel et al. have also reported that GPT-4o tends to produce longer responses than other models, without improving content quality^6,13^. Therefore, incorporating flexible response options, such as concise summaries or detailed answers, into LLMs may enhance patient comprehension.

In our study, both LLMs explicitly displayed a warning in the chat interface stating "I may make mistakes; please verify the information," and occasionally acknowledged the risk of "hallucination" (generating fabricated content). A recent systematic review by Sallam et al. highlighted the potential of LLMs to contribute to an infodemic in healthcare by producing non-evidence–based information^14^. The fact that inaccurate or incomplete information can negatively influence patients’ diagnostic and therapeutic decisions is well-established in the literature^15^. In parallel with this, in more than half of the questions they answered, the models included statements such as "consult your doctor" or "your physician knows best." The co-occurrence of "I may make mistakes" and "consult your physician" warnings can be interpreted as an inherent self-caution by LLMs, indicating that they should be used in healthcare only as supportive tools and with appropriate caution.

Although this study is among the pioneering works evaluating LLM responses on carotid artery disease and carotid endarterectomy through expert assessment, it has certain limitations. The evaluations were based on the individual scores of four cardiovascular surgeons, rendering the results subjective. As the patient perspective was not included, how responses would be perceived by users is unclear. The use of a question set prepared in Turkish and originating from Turkey may limit the generalizability of the findings to other languages and cultures. Furthermore, given that LLMs are continuously updated, the results obtained may change over time. Larger, multilingual studies with patient participation would help address these limitations.

## CONCLUSION

LLM-based chatbots have secured an important place in modern healthcare communication by offering personalized and rapid access to information. Our study demonstrated that, in answering patient questions regarding carotid artery disease and carotid endarterectomy, GPT-4o outperformed Gemini for accuracy, although both models exhibited a comparable overall "good" level of performance. Explicit "I may make mistakes" warnings in their chat interfaces underscore the need to view these tools as supportive rather than definitive resources. While LLMs may serve as helpful aids in patient education, these findings should be interpreted with caution, given the study's limited sample size, language, and cultural context. Ultimately, the final authority in clinical decision-making remains with the physician, and users should always verify the information they obtain with their doctors.

## Data Availability

The datasets generated and/or analyzed during the current study are available from the corresponding author upon reasonable request.
