# ERRATUM: Expert evaluation of GPT-4o and Gemini responses to patient questions on carotid endarterectomy

**DOI:** 10.1590/1806-9282.20251453-ER

**Published:** 2026-07-31

In the manuscript: Rahman ÖF, Özbakkaloğlu A, Arslangilay M, Daylan A, Keleş E, Bozkurt ÖT, et al. Expert evaluation of GPT-4o and Gemini responses to patient questions on carotid endarterectomy. Rev Assoc Med Bras. 2026;72(2):e20251453. https://doi.org/10.1590/1806-9282.20251453

**Where it reads:**

AŞahin Bozok

**It should read:**

Şahin Bozok

